# EWAS Open Platform 2026: a deeply integrated resource for epigenome-wide association studies

**DOI:** 10.1093/nar/gkaf1155

**Published:** 2025-11-17

**Authors:** Fei Yang, Zhuang Xiong, Wenting Zong, Demian Kong, Bixia Tang, Xupeng Chen, Yaoke Wei, Xiangyu Yu, Yiran Zhang, Dong Zou, Zhang Zhang, Yiming Bao, Rujiao Li

**Affiliations:** National Genomics Data Center, China National Center for Bioinformation, Beijing 100101, China; Beijing Institute of Genomics, Chinese Academy of Sciences, Beijing 100101, China; Interdisciplinary Institute for Medical Engineering, Fuzhou University, Fuzhou 350002, China; National Genomics Data Center, China National Center for Bioinformation, Beijing 100101, China; Beijing Institute of Genomics, Chinese Academy of Sciences, Beijing 100101, China; National Genomics Data Center, China National Center for Bioinformation, Beijing 100101, China; Beijing Institute of Genomics, Chinese Academy of Sciences, Beijing 100101, China; University of Chinese Academy of Sciences, Beijing 100049, China; National Genomics Data Center, China National Center for Bioinformation, Beijing 100101, China; Beijing Institute of Genomics, Chinese Academy of Sciences, Beijing 100101, China; Interdisciplinary Institute for Medical Engineering, Fuzhou University, Fuzhou 350002, China; National Genomics Data Center, China National Center for Bioinformation, Beijing 100101, China; Beijing Institute of Genomics, Chinese Academy of Sciences, Beijing 100101, China; University of Chinese Academy of Sciences, Beijing 100049, China; National Genomics Data Center, China National Center for Bioinformation, Beijing 100101, China; Beijing Institute of Genomics, Chinese Academy of Sciences, Beijing 100101, China; University of Chinese Academy of Sciences, Beijing 100049, China; National Genomics Data Center, China National Center for Bioinformation, Beijing 100101, China; Beijing Institute of Genomics, Chinese Academy of Sciences, Beijing 100101, China; University of Chinese Academy of Sciences, Beijing 100049, China; National Genomics Data Center, China National Center for Bioinformation, Beijing 100101, China; Beijing Institute of Genomics, Chinese Academy of Sciences, Beijing 100101, China; National Genomics Data Center, China National Center for Bioinformation, Beijing 100101, China; Beijing Institute of Genomics, Chinese Academy of Sciences, Beijing 100101, China; University of Chinese Academy of Sciences, Beijing 100049, China; National Genomics Data Center, China National Center for Bioinformation, Beijing 100101, China; Beijing Institute of Genomics, Chinese Academy of Sciences, Beijing 100101, China; University of Chinese Academy of Sciences, Beijing 100049, China; National Genomics Data Center, China National Center for Bioinformation, Beijing 100101, China; Beijing Institute of Genomics, Chinese Academy of Sciences, Beijing 100101, China

## Abstract

Epigenome-wide association studies (EWAS) has become an indispensable approach for elucidating the epigenetic basis of complex traits. EWAS Open Platform (https://ngdc.cncb.ac.cn/ewas/) includes three main components: EWAS Atlas (curated associations from publications), EWAS Data Hub (normalized DNA methylation array data), and EWAS Toolkit (one-stop analysis services). Here, we present a new release of EWAS Open Platform with the following significant updates and enhancements: (i) Expanded information: EWAS Atlas houses over 800 000 associations and incorporates an additional 17 000 curated causal relationships. EWAS Data Hub contains >180 000 batch-corrected samples, which incorporate data from newly added Illumina MethylationEPIC v2.0 BeadChip (935K) as well as trait-specific methylation profiles. EWAS Toolkit now provides an online batch correction tool and an interactive epigenetic causal network. (ii) Enhanced interoperability: data, knowledge, and toolkit are fully interconnected through a unified retrieval, offering integrated summaries and visualization capabilities. (iii) Artificial intelligence (AI)-based service: the platform is newly equipped with an AI-assisted question-answering service, allowing users to interactively explore EWAS-related questions and generate tailored insights. Taken together, EWAS Open Platform has undergone a significant upgrade across data resources, analytical tools, and service functionalities, offering more advanced support for unraveling complex molecular mechanisms from an epigenomic perspective.

## Graphical Abstract

**Graphical Abstract:**
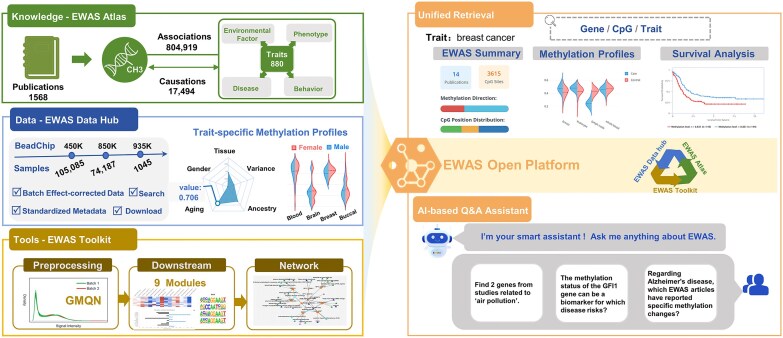

## Introduction

Epigenome-wide association studies (EWAS) has emerged as a pivotal approach for elucidating the epigenetic basis of complex traits, including diseases [1–6]. The rapidly expanding volume of EWAS research and data enables in-depth integration and mining for epigenetic biomarker discovery and regulatory mechanism exploration. However, these large-scale data resources present several significant challenges: data are often dispersed and lack standardized formats, batch effects remain inadequately addressed, knowledge is fragmented with uncertain reliability, and analytical workflows are complex and cumbersome, together highlighting the urgent need for more systematic and efficient solutions [7]. To address this need, EWAS Open Platform (https://ngdc.cncb.ac.cn/ewas/) has been developed, including three components: EWAS Atlas [8], which focuses on the literature curation of EWAS associations; EWAS Data Hub [9], which is dedicated to the integration and normalization of large-scale methylation datasets; and EWAS Toolkit [10], which offers a suite of user-friendly, one-stop online tools for EWAS analysis. Ultimately, these components were unified into a comprehensive and scalable resource, with continuous updates and upgrades to support the full spectrum of EWAS [11–19].

To consistently provide up-to-date and comprehensive services, the updated EWAS Open Platform further strengthens the interoperability across data, knowledge, and toolkits (Table 1). EWAS Atlas adds a curated causal relationships module for more precise insights into epigenetic mechanisms. EWAS Data Hub expands data diversity, feature coverage, and trait-specific DNA methylation profiles. EWAS Toolkit now provides upstream batch correction for DNA methylation arrays, including the latest Illumina MethylationEPIC v2.0 BeadChip (935K), and downstream multi-omics causal networks. Beyond the three core components, the most transformative advancement in this release is the unification of data and knowledge through an integrated, visualized retrieval. Leveraging an artificial intelligence (AI)-based Q&A assistant, users can interactively explore EWAS-related queries and obtain personalized insights from the platform. This innovation positions the platform as a deeply integrated system that tightly links data access with biological interpretation, thereby offering researchers a more coherent, intelligent, and efficient resource for epigenetic investigations.

**Table 1.** Main updates of EWAS Open Platform

		EWAS Open Platform (2022)	EWAS Open Platform (2026)
**Knowledge**			
Publications		910	1568
Associations		617 018	806 860
Causations	Total	NA	17 494
	Methylation→trait	NA	3836
	Trait→methylation	NA	45
	Methylation→expression	NA	10 557
	Trait→trait	NA	1193
Traits		618	880
Studies		1437	1908
Cohorts		3382	3989
**Data**			
Samples	Total	115 852	180 317
	935K^a^	NA	1045
	850K^b^	20 923	74 187
	450K^c^	94 929	105 085
Tissues or cells		1118	1413
Diseases		528	842
Fields		242	296
Trait-specific methylation profiles		450K^c^	450K^c^,850K^b^
**Toolkit**			
Causation network		NA	Yes
Batch effect correction—GMQN^d^		NA	Yes
**Unified retrieval**		NA	Yes
**AI- based Q&A assistant**		NA	Yes

a Illumina MethylationEPIC v2.0 BeadChip.

b Illumina MethylationEPIC v1.0 BeadChip.

c Illumina HumanMethylation450 BeadChip.

d Gaussian mixture quantile normalization.

## New features and updates

### Knowledge—EWAS Atlas

**Figure 1.**
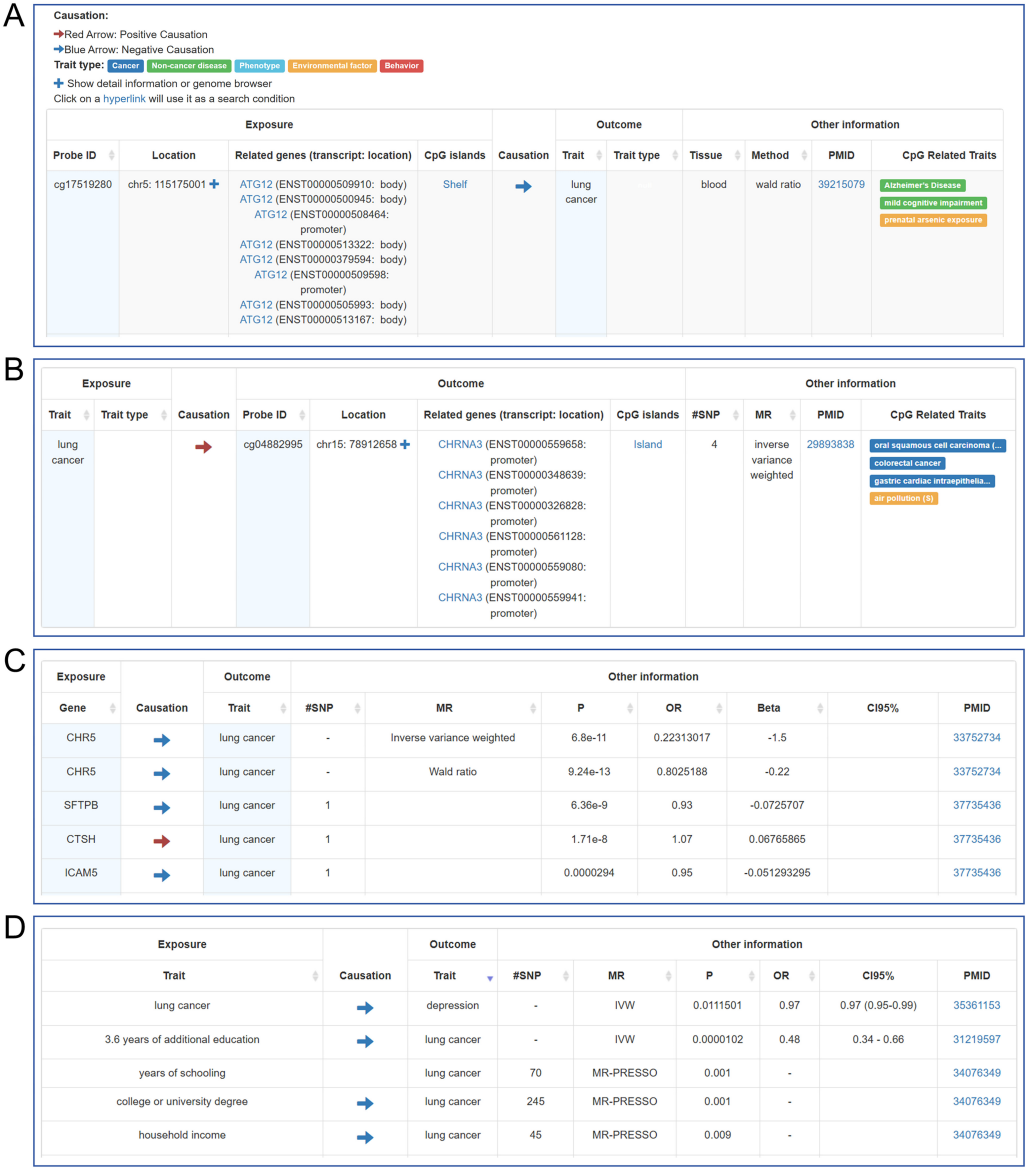
Retrieval results from the causal knowledge module using lung cancer as an example. (**A**) Causal relationships from DNA methylation to lung cancer. (**B**) Causal relationships from lung cancer to DNA methylation. (**C**) Causal relationships from gene expression to lung cancer. (**D**) Causal relationships between lung cancer and other traits.

To move beyond simple associations toward causal inference [20], the updated EWAS Atlas systematically integrates curated causal knowledge into its previously association-centric framework, thereby enabling more precise mechanistic insights and facilitating the discovery of potential intervention targets. This module incorporates causal relationships derived from Mendelian randomization studies [21–23], including 3836 methylation-to-trait, 45 trait-to-methylation, 10 555 methylation-to-expression, 1862 expression-to-trait, and 1193 trait-to-trait associations curated from publications. To facilitate efficient data exploration, the platform provides query functionality that allows searching by traits, methylation sites, and genes. Queries for diseases such as lung cancer reveal potential causal associations among methylation sites, gene expression events, and related traits (Fig. 1). This functionality enables researchers to assess whether epigenetic variations are linked with specific traits and to investigate potential causal mechanisms underlying disease, thereby supporting the identification of biomarkers, intervention targets, and therapeutic candidates. Meanwhile, we have continuously tracked new publications on association studies, adding 189 142 trait–methylation associations and expanding the coverage to 262 new traits, including environmental factors, behaviors, phenotypes, and diseases. Overall, EWAS Atlas now hosts 806 860 high-quality EWAS associations and 17 494 causal links derived from 1568 publications, representing a >70% increase compared to the previous release. These span 248 tissues and cell types, 880 traits, and over 35 000 genes, providing an unprecedented breadth of coverage (Table 1).

### Data—EWAS Data Hub

To keep pace with the latest technological advances in DNA methylation and enable high-resolution, trait-specific epigenomic analysis, EWAS Data Hub has now incorporated data from the newly released 935K array, alongside existing Illumina HumanMethylation450 BeadChip (450K) and Illumina MethylationEPIC v1.0 BeadChip (850K) arrays. It features harmonized methylation profiles for 180 317 samples from public sources, spanning 1413 tissues/cell types and 842 diseases. Notably, all newly incorporated data, including the 935K samples, underwent Gaussian mixture quantile normalization (GMQN)-based batch effect correction [24], ensuring consistency across samples for downstream analyses. In parallel, the 850K samples have undergone substantial growth, increasing from 20 923 to 74 156 samples, representing a >3.5-fold expansion. Leveraging this expanded data, we newly constructed comprehensive trait-specific methylation profiles at 898 918 CpG sites, characterizing distinct methylation patterns across tissues, age groups, sex, ethnicity, BMI, 76 cancer types, and 60 non-cancer diseases (Table 1).

### Tools—EWAS Toolkit

To assist users in performing batch effect correction on their own methylation data and to enable seamless integration or comparative analyses with large-scale data in EWAS Data Hub, we have launched a one-click online batch effect correction tool based on GMQN [24], which supports data from not only 450K and 850K but also 935K arrays, thereby significantly enhancing its applicability and utility. The service enables users to submit data through multiple approaches, including uploading m/um files or directly inputting Gene Expression Omnibus (GEO) [25, 26] dataset accession numbers, thereby offering a more flexible and convenient solution for batch effect correction. Furthermore, to enable users to visualize disease-related molecular interaction networks and explore directed causal relationships, we incorporated a multilayer user-interactive causal network using Cytoscape plugins, systematically integrating causal relationships at epigenomic, transcriptomic, and trait levels, thereby providing novel insights into molecular mechanisms underlying diseases. The network supports multidimensional queries based on traits, genes, and CpG sites, and allows users to flexibly adjust node ranges to generate directed causal maps centered on the queried entities. Taken together, EWAS Toolkit now offers 12 tools, delivering comprehensive and streamlined services that cover the entire analytical workflow, from upstream data preprocessing to downstream functional annotation and exploration.

### Unified retrieval

**Figure 2.**
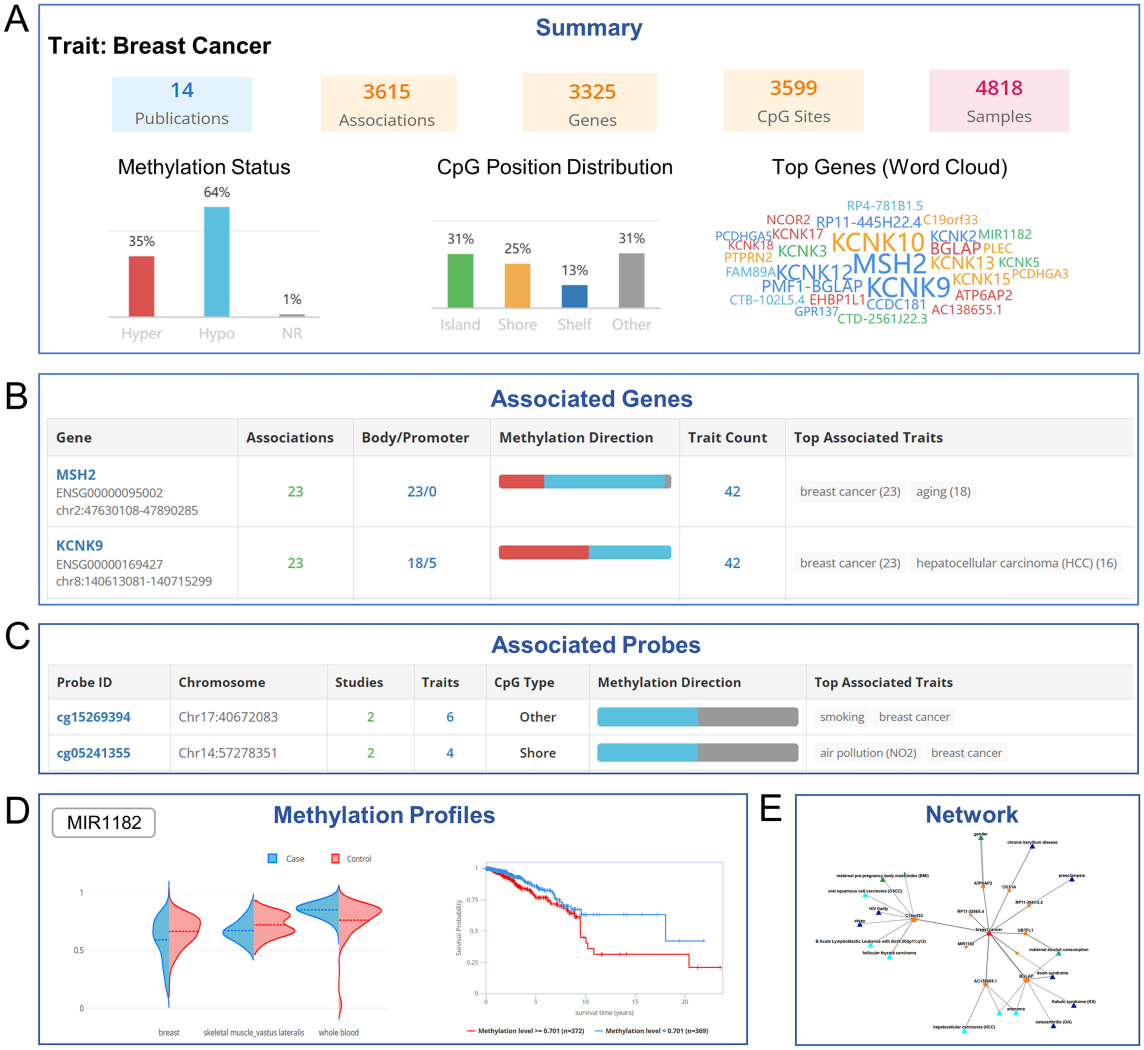
Unified retrieval results from EWAS Open Platform using breast cancer as an example. (**A**) Overview of EWAS publications, datasets, and associated methylation sites related to breast cancer. (**B**) Characteristics of representative methylated genes associated with breast cancer. (**C**) Features of representative methylation sites associated with breast cancer. (**D**) Differential methylation patterns between breast cancer and normal samples across different tissue types and their association with patient survival. (**E**) Epigenetic association network related to breast cancer.

To address the fragmentation of data, knowledge, and toolkit on EWAS Open Platform and to enable efficient, integrated access, we developed a seamless cross-module unified retrieval that combines quantitative summaries with interactive visualizations. This interface leverages a unified application programming interface (API), integrates heterogeneous data through semantic parsing, and employs standardized data modeling, ensuring consistency, interoperability, and robust query support across diverse data types. Users can query by traits, genes, or CpG probe IDs, with results delivered through concise summaries and intuitive visual outputs. For instance, a query for “breast cancer” returns comprehensive quantitative summaries, including the number and categories of methylation sites and genes reported in publications as associated with breast cancer within EWAS Atlas, along with corresponding number of sample from EWAS Data Hub. Additional outputs include the proportions of hyper- and hypomethylated sites, the genomic distribution of CpG sites, and a word cloud highlighting the most frequently reported differentially methylated genes (Fig. 2A). Users can further examine detailed information on representative methylation sites and genes associated with breast cancer (Fig. 2B and C). Moreover, the platform illustrates the methylation patterns of these genes in breast cancer and their associations with patient survival, based on analyses from EWAS Data Hub (Fig. 2D), and presents the epigenetic association network related to breast cancer (Fig. 2E). Embedded links provide seamless navigation and cross-module exploration of related content.

### AI-based Q&A assistant

**Figure 3.**
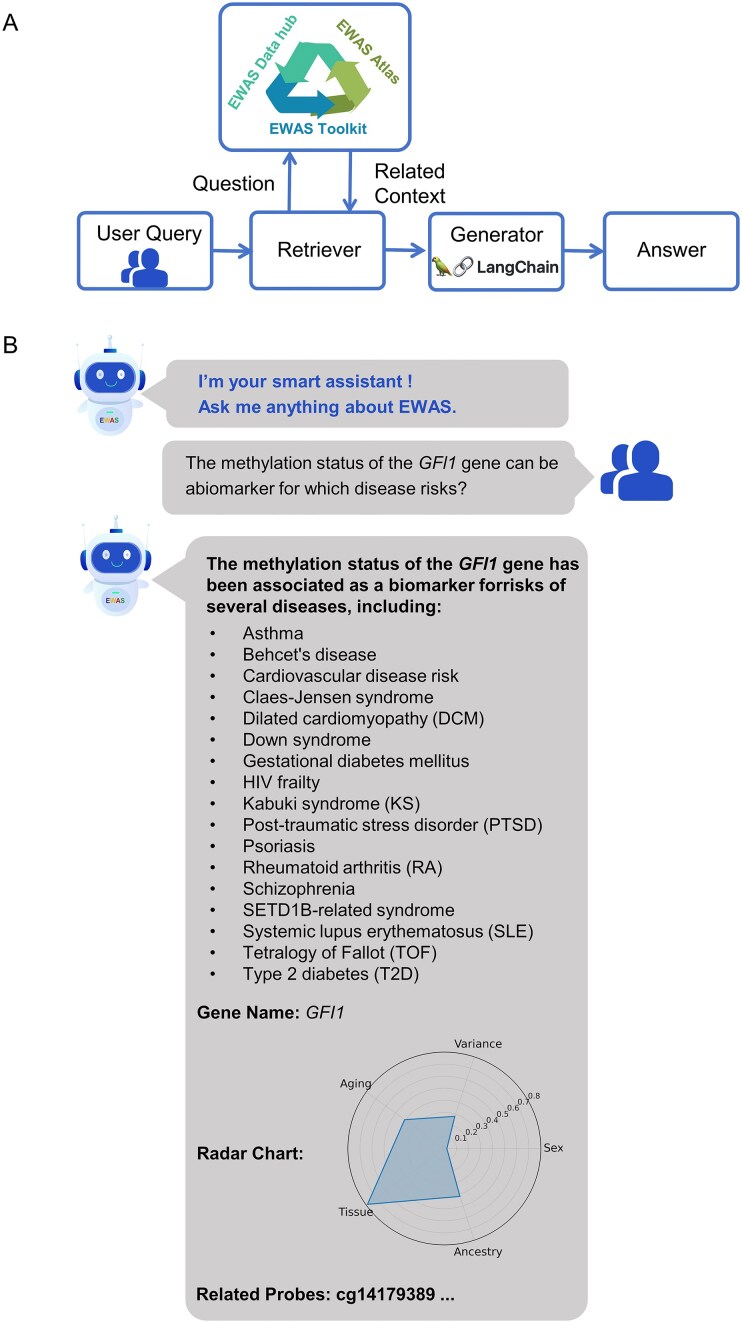
The AI-based question-answering assistant of EWAS Open Platform. (**A**) The architecture of AI-assisted Q&A assistant. (**B**) Example output for the query: “The methylation status of the *GFI1* gene can be a biomarker for which disease risks?”

To enable researchers to access epigenetic information on complex traits including diseases in a more flexible and intuitive manner, we developed an intelligent agent-based question-answering assistant within EWAS Open Platform, using the LangChain and LangGraph frameworks [27, 28] along with Vue3 [29] (Fig. 3A). Technically, the assistant implements a multistage text-to-SQL pipeline, including intelligent entity recognition, context-aware SQL generation, data querying and structuring, and enrichment and secondary processing, as well as final delivery to the frontend. Retrieved data are displayed in the user interface for in-depth exploration and analysis. Meanwhile, the processed results are passed to a large language model, which produces clear, accurate, and informative natural language answers, effectively transforming unstructured queries into actionable epigenetic insights through intelligent reasoning.

The assistant supports conversational queries in natural language and automatically returns structured answers derived from integrated EWAS resources, covering disease associations, methylation patterns, and multidimensional factors such as age, sex, ethnicity, and tissue. For example, when asked “The methylation status of the *GFI1* gene can be a biomarker for which disease risks?,” the assistant compiles a panel summarizing diseases associated with *GFI1* and its involvement in biological processes. In addition, the assistant provides a radar chart of *GFI1*–trait associations and visualizes methylation sites and their characteristics (Fig. 3B). By linking unstructured queries to comprehensive EWAS resources, the AI-based assistant offers researchers, including those who are not specialists, a more direct and flexible way to explore complex epigenetic information and insights. It organizes and presents these insights into intuitive visualizations and structured summaries, facilitating interpretation, supporting the identification of potential associations, and leveraging intelligent reasoning to guide new directions in epigenetic research.

## Discussion and future developments

EWAS Open Platform, as one of the essential resources within National Genomics Data Center, China National Center for Bioinformation [30, 31], continues to provide researchers with an integrated suite of data, knowledge, and toolkit to support EWAS. Furthermore, the high-quality datasets available through this platform have already facilitated the training of several AI models [32–34]. This update delivers significant advancements across multiple dimensions, including the targeted expansion of causal knowledge, the enhancement of data profiles, the integration of upstream and downstream analytical tools, AI-based query capabilities, and the implementation of comprehensive, interactive data-knowledge retrieval functionalities. Collectively, these improvements brings EWAS Open Platform closer to its vision of serving as a robust and integrative resource for advancing epigenomic research.

Looking ahead, we plan to further embed AI within the epigenetic knowledge curation framework and to establish more automated pipelines for data analysis, thereby enhancing the efficiency and rigor of knowledge discovery. Concurrently, EWAS Open Platform will not only strengthen its connections with resources, such as MethBank [35–37], scMethBank [5], and DiseaseMeth [38], but also enhance data quality, enrich annotation content, and extend its scope beyond DNA methylation to encompass a broader spectrum of epigenetic associations [39–46], thereby expanding the depth and breadth of integrative studies and empowering AI-driven analyses. In terms of implementation, we aim to develop end-to-end, sample-selectable workflows alongside user-friendly online tools for differential methylation analysis and functional annotation. In parallel, the integration of EWAS with genome-wide association study (GWAS) and transcriptome-wide association study (TWAS) will be strengthened to foster multi-omics synergy and to address critical scientific challenges in population health research [12, 47–50]. Collectively, these improvements will provide the research community with a versatile, interoperable, and sustainable platform, further enabling AI applications, accelerating epigenomic discovery, and elucidating the complex mechanisms underlying human health and disease.

## Data Availability

EWAS Open Platform is a comprehensive resource for EWAS. All data, knowledge, and tools are freely available at https://ngdc.cncb.ac.cn/ewas/. Any queries, comments, and suggestions on EWAS Open Platform can be provided by email via ewas-user@big.ac.cn.
